# Supplementary material for: New estimations of child marriage: Evidence from 98 low- and middle-income countries
Source: PLoS One. 2021 Oct 27;16(10):e0258378. doi: 10.1371/journal.pone.0258378 (PMC8550406; doi:10.1371/journal.pone.0258378)
Supplement: S1 Table — (DOCX) [file pone.0258378.s001.docx]

| Country | Reference year | Data Source | Married before 15 | Married before 18 | Direct approach | Survival analysis approach | Direct approach | Survival analysis approach |
| --- | --- | --- | --- | --- | --- | --- | --- | --- |
|  |  |  | Among women age 20-24 (%) | | Estimated number of girls marrying before age 15 (000) | | Estimated number of girls marrying before age 18 (000) | |
| Albania | 2018 | DHS 2017-2018 | 1.4 | 11.8 | 0.2 | 0.2 | 2.3 | 1.2 |
| Algeria | 2019 | MICS 2018-2019 | 0.0 | 3.8 | 0.1 | 0.5 | 10.3 | 10.0 |
| Angola | 2016 | DHS 2015-2016 | 7.9 | 30.3 | 30.6 | 20.6 | 103.3 | 82.7 |
| Armenia | 2016 | DHS 2016 | 0.0 | 5.3 | 0.0 | 0.0 | 0.8 | 0.7 |
| Azerbaijan | 2006 | DHS 2006 | 0.7 | 12.2 | 0.4 | 0.3 | 7.2 | 6.5 |
| Bangladesh | 2019 | MICS 2019 | 15.5 | 51.4 | 233.9 | 112.2 | 785.5 | 551.4 |
| Barbados | 2012 | MICS 2012 | 7.7 | 29.2 | 0.1 | 0.1 | 0.5 | 0.8 |
| Belarus | 2019 | MICS 2019 | 0.1 | 4.7 | 0.1 | 0.0 | 2.0 | 0.9 |
| Belize | 2016 | MICS 2015-2016 | 6.3 | 33.5 | 0.2 | 0.1 | 1.3 | 1.1 |
| Benin | 2018 | DHS 2017-2018 | 9.4 | 30.6 | 12.7 | 6.0 | 37.8 | 28.5 |
| Bhutan | 2010 | MICS 2010 | 6.2 | 25.8 | 0.4 | 0.2 | 1.8 | 1.2 |
| Bolivia (Plurinational State of) | 2008 | DHS 2008 | 3.2 | 21.7 | 3.6 | 2.8 | 24.0 | 17.9 |
| Bosnia and Herzegovina | 2011 | MICS 2011 | 0.2 | 3.5 | 0.0 | 0.0 | 0.6 | 0.2 |
| Burkina Faso | 2010 | DHS 2010 | 10.2 | 51.6 | 24.7 | 15.1 | 112.4 | 87.9 |
| Burundi | 2017 | DHS 2016-2017 | 2.8 | 19.0 | 3.7 | 0.3 | 22.1 | 8.3 |
| Cambodia | 2014 | DHS 2014 | 1.9 | 18.5 | 2.9 | 2.2 | 26.6 | 26.8 |
| Cameroon | 2018 | DHS 2018 | 10.7 | 29.8 | 32.4 | 17.7 | 82.0 | 69.9 |
| Central African Republic | 2019 | MICS 2018-2019 | 25.8 | 61.0 | 16.3 | 11.1 | 35.3 | 28.6 |
| Chad | 2019 | MICS 2019 | 24.2 | 60.6 | 48.0 | 18.6 | 108.3 | 64.7 |
| Colombia | 2015 | DHS 2015 | 4.9 | 23.4 | 19.5 | 14.7 | 99.2 | 75.9 |
| Comoros | 2012 | DHS 2012 | 10.0 | 31.6 | 0.9 | 0.6 | 2.7 | 2.1 |
| Congo | 2015 | MICS 2014-2015 | 6.9 | 27.3 | 4.2 | 2.8 | 14.9 | 12.7 |
| Costa Rica | 2018 | MICS 2018 | 2.0 | 17.1 | 0.7 | 0.6 | 6.1 | 4.3 |
| Cote d'Ivoire | 2016 | MICS 2016 | 7.0 | 27.0 | 21.0 | 14.2 | 77.0 | 60.6 |
| Cuba | 2019 | MICS 2019 | 4.8 | 29.4 | 2.9 | 3.2 | 18.7 | 12.0 |
| Democratic Republic of the Congo | 2018 | MICS 2017-2018 | 8.4 | 29.1 | 86.1 | 48.6 | 263.1 | 218.1 |
| Dominican Republic | 2013 | DHS 2013 | 10.2 | 36.5 | 9.7 | 9.7 | 34.2 | 29.4 |
| El Salvador | 2014 | MICS 2014 | 5.8 | 25.5 | 3.3 | 3.3 | 15.1 | 12.9 |
| Eswatini | 2014 | MICS 2014 | 0.8 | 5.3 | 0.1 | 0.1 | 0.7 | 0.6 |
| Ethiopia | 2016 | DHS 2016 | 14.1 | 40.3 | 186.3 | 75.9 | 511.3 | 332.2 |
| Gabon | 2012 | DHS 2012 | 5.6 | 21.9 | 1.1 | 0.8 | 4.1 | 2.8 |
| Gambia | 2018 | MICS 2018 | 7.5 | 25.7 | 2.0 | 1.3 | 6.4 | 5.4 |
| Georgia | 2018 | MICS 2018 | 0.3 | 13.9 | 0.1 | 0.4 | 2.8 | 2.2 |
| Ghana | 2018 | MICS 2017-2018 | 5.0 | 19.3 | 16.0 | 4.3 | 58.1 | 34.2 |
| Guatemala | 2015 | DHS 2014-2015 | 6.2 | 29.5 | 11.9 | 10.1 | 56.2 | 47.4 |
| Guinea | 2018 | DHS 2018 | 17.0 | 46.5 | 26.8 | 16.7 | 67.9 | 53.5 |
| Guinea-Bissau | 2019 | MICS 2018-2019 | 8.1 | 25.7 | 1.8 | 0.6 | 5.2 | 3.8 |
| Guyana | 2020 | MICS 2019-2020 | 6.3 | 32.3 | 0.5 | 0.5 | 2.4 | 2.4 |
| Haiti | 2017 | DHS 2016-2017 | 2.1 | 14.9 | 2.5 | 1.8 | 16.8 | 10.2 |
| Honduras | 2012 | DHS 2011-2012 | 7.6 | 33.6 | 7.7 | 8.1 | 34.3 | 31.4 |
| India | 2016 | DHS 2015-2016 | 5.4 | 25.3 | 645.0 | 221.9 | 2986.6 | 1904.5 |
| Indonesia | 2017 | DHS 2017 | 2.0 | 16.3 | 44.1 | 26.0 | 370.7 | 260.8 |
| Iraq | 2018 | MICS 2018 | 7.2 | 27.9 | 30.0 | 18.9 | 111.0 | 91.3 |
| Jamaica | 2011 | MICS 2011 | 1.4 | 7.9 | 0.3 | 0.0 | 1.9 | 0.9 |
| Kazakhstan | 2015 | MICS 2015 | 0.2 | 7.0 | 0.2 | 0.0 | 7.1 | 5.9 |
| Kenya | 2014 | DHS 2014 | 4.4 | 22.9 | 27.6 | 10.6 | 133.5 | 86.7 |
| Kiribati | 2019 | MICS 2018-2019 | 2.4 | 18.4 | 0.0 | 0.0 | 0.2 | 0.1 |
| Kyrgyzstan | 2018 | MICS 2018 | 0.3 | 12.9 | 0.2 | 0.1 | 6.2 | 3.1 |
| Lao People's Democratic Republic | 2017 | MICS 2017 | 7.1 | 32.7 | 5.1 | 4.2 | 22.6 | 18.9 |
| Lesotho | 2018 | MICS 2018 | 1.0 | 16.4 | 0.2 | 0.2 | 3.6 | 3.4 |
| Liberia | 2013 | DHS 2013 | 8.8 | 35.9 | 5.1 | 2.2 | 19.0 | 10.7 |
| Madagascar | 2018 | MICS 2018 | 12.7 | 40.3 | 39.8 | 33.9 | 118.6 | 111.2 |
| Malawi | 2016 | DHS 2015-2016 | 9.0 | 42.1 | 21.1 | 8.6 | 88.3 | 64.6 |
| Maldives | 2017 | DHS 2016-2017 | 0.0 | 2.2 | 0.0 | 0.0 | 0.1 | 0.0 |
| Mali | 2018 | DHS 2018 | 15.9 | 53.7 | 38.6 | 35.8 | 113.6 | 112.5 |
| Mauritania | 2015 | MICS 2015 | 17.8 | 37.0 | 8.7 | 6.2 | 16.7 | 15.1 |
| Mexico | 2015 | MICS 2015 | 3.8 | 26.1 | 41.8 | 32.2 | 288.6 | 236.9 |
| Mongolia | 2018 | MICS 2018 | 0.9 | 12.0 | 0.2 | 0.0 | 2.5 | 1.0 |
| Montenegro | 2018 | MICS 2018 | 1.9 | 5.8 | 0.1 | 0.0 | 0.2 | 0.1 |
| Mozambique | 2011 | DHS 2011 | 14.3 | 48.2 | 54.0 | 39.1 | 166.7 | 172.5 |
| Myanmar | 2016 | DHS 2015-2016 | 1.9 | 16.0 | 9.5 | 5.2 | 81.3 | 74.8 |
| Namibia | 2013 | DHS 2013 | 1.6 | 6.9 | 0.4 | 0.2 | 1.7 | 1.7 |
| Nepal | 2019 | MICS 2019 | 7.9 | 32.8 | 24.3 | 8.1 | 105.7 | 64.5 |
| Niger | 2012 | DHS 2012 | 28.0 | 76.3 | 79.2 | 67.4 | 188.5 | 179.7 |
| Nigeria | 2018 | DHS 2018 | 15.7 | 43.4 | 362.1 | 197.1 | 901.7 | 630.6 |
| Panama | 2013 | MICS 2013 | 6.8 | 26.4 | 2.4 | 1.6 | 9.2 | 7.2 |
| Papua New Guinea | 2018 | DHS 2016-2018 | 8.0 | 27.3 | 7.4 | 3.4 | 24.0 | 14.9 |
| Paraguay | 2016 | MICS 2016 | 3.6 | 21.6 | 2.3 | 2.4 | 13.9 | 13.9 |
| Peru | 2012 | DHS 2012 | 2.5 | 19.1 | 6.5 | 5.0 | 47.4 | 35.6 |
| Philippines | 2017 | DHS 2017 | 2.2 | 16.5 | 23.0 | 11.7 | 165.6 | 107.3 |
| Republic of Moldova | 2012 | MICS 2012 | 0.4 | 12.2 | 0.1 | 0.0 | 2.4 | 2.1 |
| Republic of North Macedonia | 2019 | MICS 2018-2019 | 0.3 | 7.5 | 0.0 | 0.0 | 0.9 | 0.5 |
| Rwanda | 2015 | DHS 2014-2015 | 0.4 | 6.8 | 0.5 | 0.0 | 9.0 | 4.7 |
| Saint Lucia | 2012 | MICS 2012 | 3.7 | 24.0 | 0.0 | 0.0 | 0.3 | 0.2 |
| Sao Tome and Principe | 2019 | MICS 2019 | 5.4 | 28.0 | 0.1 | 0.1 | 0.7 | 0.4 |
| Senegal | 2014 | DHS 2014 | 8.7 | 32.3 | 16.3 | 11.5 | 54.6 | 50.0 |
| Serbia | 2019 | MICS 2019 | 1.2 | 5.5 | 0.6 | 0.5 | 2.7 | 1.5 |
| Sierra Leone | 2019 | DHS 2019 | 8.6 | 29.6 | 8.0 | 3.2 | 25.1 | 15.7 |
| South Africa | 2016 | DHS 2016 | 0.9 | 3.6 | 4.8 | 2.0 | 17.1 | 18.4 |
| South Sudan | 2010 | SHHS 2010 | 8.9 | 51.5 | 11.1 | 6.8 | 59.6 | 51.0 |
| State of Palestine | 2020 | MICS 2019-2020 | 0.7 | 13.4 | 0.4 | 0.2 | 6.7 | 4.6 |
| Sudan | 2014 | MICS 2014 | 11.9 | 34.2 | 58.9 | 29.2 | 158.5 | 112.6 |
| Suriname | 2018 | MICS 2018 | 8.8 | 36.0 | 0.4 | 0.7 | 1.7 | 2.3 |
| Tajikistan | 2017 | DHS 2017 | 0.1 | 8.7 | 0.1 | 0.0 | 6.7 | 3.8 |
| Thailand | 2019 | MICS 2019 | 3.0 | 20.2 | 12.5 | 11.3 | 87.6 | 60.3 |
| Timor-Leste | 2016 | DHS 2016 | 2.6 | 14.9 | 0.4 | 0.2 | 2.2 | 1.6 |
| Togo | 2017 | MICS 2017 | 6.4 | 24.8 | 6.1 | 1.8 | 21.3 | 13.4 |
| Tonga | 2019 | MICS 2019 | 0.4 | 10.1 | 0.0 | 0.0 | 0.1 | 0.1 |
| Tunisia | 2018 | MICS 2018 | 0.0 | 1.5 | 0.0 | 0.0 | 1.1 | 0.4 |
| Turkmenistan | 2019 | MICS 2019 | 0.2 | 6.1 | 0.1 | 0.0 | 2.9 | 1.6 |
| Uganda | 2016 | DHS 2016 | 7.3 | 34.0 | 40.8 | 16.4 | 172.3 | 141.6 |
| Ukraine | 2012 | MICS 2012 | 0.1 | 9.1 | 0.2 | 1.3 | 16.8 | 11.3 |
| United Republic of Tanzania | 2016 | DHS 2015-2016 | 5.2 | 30.5 | 35.8 | 24.4 | 187.5 | 189.4 |
| Uruguay | 2013 | MICS 2013 | 0.7 | 24.6 | 0.2 | 0.3 | 6.0 | 4.0 |
| Viet Nam | 2014 | MICS 2014 | 0.9 | 10.6 | 5.9 | 5.0 | 65.7 | 64.2 |
| Yemen | 2013 | DHS 2013 | 9.4 | 31.9 | 30.9 | 11.2 | 98.3 | 63.7 |
| Zambia | 2018 | DHS 2018 | 5.2 | 29.0 | 11.6 | 5.1 | 59.6 | 39.1 |
| Zimbabwe | 2019 | MICS 2019 | 5.4 | 33.7 | 9.5 | 3.3 | 53.6 | 43.4 |
